# Norswertianolin Promotes Cystathionine γ-Lyase Activity and Attenuates Renal Ischemia/Reperfusion Injury and Hypertension

**DOI:** 10.3389/fphar.2021.677212

**Published:** 2021-07-14

**Authors:** Yaping Niu, Congkuo Du, Changting Cui, Haizeng Zhang, Yue Deng, Jun Cai, Zhenzhen Chen, Bin Geng

**Affiliations:** ^1^ Hypertension Center, Fuwai Hospital, Chinese Academy of Medical Sciences and Peking Union Medical College, State Key Laboratory of Cardiovascular Disease, National Center for Cardiovascular Diseases, Beijing, China; ^2^ Institute of Hypoxia Medicine, Wenzhou Medical University, Wenzhou, China

**Keywords:** cystathionine gamma-lyase, hydrogen sulfide, norswertianolin, renal ischemia/reperfusion, hypertension

## Abstract

Cystathionine gamma-lyase (CSE)/hydrogen sulfide (H_2_S) plays a protective role in cardiovascular diseases including hypertension and ischemia/reperfusion (I/R) injury. This study was aimed to screen natural small molecule compounds that activate CSE activity and then evaluate its effect(s) on kidney I/R injury and hypertension. Applying computer molecular docking technology, we screened the natural small molecule compound norswertianolin (NW)-specific binding to CSE. Using the microscale thermophoresis technology, we confirmed that the Leu68 site was the essential hydrogen bond site of NW binding to CSE. NW supplementation significantly increased CSE expression and its activity for H_2_S generation both *in vivo* and *in vitro*. In the model of acute and long-term kidney I/R injury, NW pretreatment dramatically attenuated kidney damage, associated with decreasing blood urea nitrogen (BUN), serum creatinine (Cr) level, reactive oxygen species (ROS) production, and cleaved caspase 3 expression. In spontaneously hypertensive rats (SHRs), NW treatment also lowered blood pressure, the media/lumen ratio of the femoral artery, and the mRNA level of inflammatory cytokines. In conclusion, NW acts as a novel small molecular chemical compound CSE agonist, directly binding to CSE, heightening CSE generation–H_2_S activity, and then alleviating kidney I/R injury and hypertension. NW has a potential therapeutic merit for cardiovascular diseases.

## Introduction

More and more studies demonstrate that hydrogen sulfide (H_2_S), as a third gasotransmitter, exhibits critical physiological and pathophysiological roles in cerebrovascular diseases (Paul and Snyder, 2018), pulmonary vascular diseases (Chunyu et al., 2003), and cardiovascular diseases (Yu et al., 2014; Pan et al., 2017). Hydrogen sulfide is metabolized from cysteine by enzymatic reaction in the presence of cystathionine-*γ*-lyase (CSE), cystathionine-*β*-synthetase (CBS), and 3-mercaptopyruvate sulfurtransferase (3-MST). CBS is present mostly in the central nervous system, while CSE is mainly expressed in the cardiovascular system (Predmore et al., 2012). In organs that expressed both CSE and CBS, CSE contributed 90% H_2_S production (Singh and Banerjee, 2011). Therefore, it is a promising therapeutic strategy to supplement H_2_S-releasing donors or CSE agonists.

In recent years, remarkable progress has been made in the field of H_2_S donors’ identification and synthesis. Early H_2_S donors such as sulfide salts (Na_2_S, NaHS), GYY4137, and 5-(4-hydroxyphenyl)-3H-1,2-dithiole-3-thione (ADT-OH) effectively promote the H_2_S level and have been widely used in scientific research (Powell et al., 2018). SG1002, a sodium polysulthionate, is a synthetic H_2_S prodrug that contains > 90% α-sulfur, has a complete phase I clinical trial for heart failure, and confirms its safety and efficacy for restoring sulfate and nitric oxide levels in heart failure patients (Polhemus et al., 2015). Recently, thiol-activated H_2_S donors, pH-controllable H_2_S donors, enzyme-dependent H_2_S-releasing donors, and other strategy H_2_S donors (mitochondrial target; hybrid drugs: ZYZ803, zofenopril, and ATB-346) also had great progress in cardiovascular diseases (Li et al., 2018). All these H_2_S donors comply with the direct supplemental H_2_S strategy and have shortcomings such as short half-life *in vivo* metabolism, potential toxicity, and poor druggability (Beltowski, 2015). The strategy-dependent drugs also increase investment costs but do not rescue endogenous H_2_S generation. In the present study, we sought to look for natural small molecule compounds as agonists of CSE, stimulating endogenous H_2_S production and release. Based on computer molecular docking technology from the Chinese Natural Products Database (CNPD), we predicted that norswertianolin (NW), a natural xanthone from *Gentianella* plants, had high binding affinity with CSE. Thus, this study was aimed to confirm NW binding to CSE and its regulation on CSE activity for H_2_S generation and its usage in cardiovascular diseases.

## Materials and Methods

### Animals and Materials

To develop the kidney I/R model, male Sprague Dawley (S-D) rats at 10–12 weeks of age were used. Male Wistar Kyoto (WKY) and spontaneously hypertensive rats (SHRs) aged 16–18 weeks were used for detecting the role of NW in hypertension. All of rats were kept in a temperature- and humidity-controlled room and had free access to tap water. All animal protocols complied with all relevant ethical regulations and were approved by the Institutional Animal Care and Use Committee, Experimental Animal Center, Fuwai Hospital, National Center for Cardiovascular Diseases, China. DMEM, DMEM/F12 culture medium, and TRIZOL reagent were from Invitrogen. Primary antibodies for β-actin (SC-47778) and Eif5 (SC-28309) were from Santa Cruz Biotechnology. The anti-CSE antibody (ab151769) was from Abcam. DL-Propargylglycine (PPG) (P7888) was from Sigma-Aldrich. NW (54954-12-0) was from Lookchem Biological Technology Co., Ltd.

### Microscale Thermophoresis Assay

Plasmid overexpression CSE and mutant CSE (Leu68 site and Asp164 site) were purchased from the biological company (Vigene, China) and cloned into pEGFP-N1 to generate new plasmids encoding CSE-GFP fusion protein. The plasmids were purified and amplified by *E. coli*. Three kinds of fusion protein plasmids were transfected into HEK-293 cells, which were continuously cultured for 24 h and expressed light green fluorescence; the cells were collected and then homogenized by RIPA buffer. The binding kinetics of NW with GFP-fused CSE was detected by microscale thermophoresis (MST, Nano Temper, Germany) (Wienken et al., 2010).

### H_2_S Production Measurement

H_2_S production was measured by the modified methylene blue method as described previously (Su et al., 2016). In detail, we prepared several custom-made 25 ml conical flasks with internal space divided into two parts. The inner ring of the conical flask was added with 0.5 ml 5% zinc acetate and a piece of filter paper (2 × 3 cm) to absorb the released H_2_S in the form of ZnS. The outer ring of the conical flask was added with 0.9 ml incubation buffer containing 100 mmol/L potassium phosphate buffer (PH 7.4), 10 mmol/L L-Cys, and 2 mmol/L pyridoxal 5′-phosphate. The rat liver, kidney, heart, and aorta were homogenized in ice-cold 50 mmol/L potassium phosphate buffer (pH 6.8) at a ratio of 10 μL/μg. 100 μL tissue homogenate was added into the outer ring, and the conical flasks were sealed and incubated in a 37°C shaking water bath for a period of time (liver for 90 min, heart and kidney for 3 h, and aorta for 6 h). 1 ml 20% trichloroacetic acid (TCA) was added into the outer ring to block the reaction and incubated for another 60 min to make sure that all hydrogen sulfide produced was absorbed. The filter paper and zinc acetate solution in the inner ring were transferred to a new tube, and 500 μL 0.2% N,N-dimethyl-*p*-phenylenediamine sulfate and 50 μL 10% ammonium ferric sulfate were added in turn. After 20 min, the absorbance at 670 nm was measured with a microplate reader.

### Cell Isolation and Culture

Primary adipocytes were isolated as we previously described (Su et al., 2016). Briefly, adipose-derived stromal cells were isolated from epididymal or inguinal subcutaneous fat pads of male rats. Fat pads were minced and digested in serum-free DMEM containing 0.8 mg/ml type I collagenase, 1% defatted BSA, 200 nM adenosine, and 25 mM HEPES (pH 7.2) for 40 min at 37°C in a water bath shaken at 120 cycles/min. The digestion mixture was filtered through 80 and 400 steel meshes to remove debris and floating adipocytes and then centrifuged at 800 rpm for 10 min. The cell pellet containing adipose precursor cells was collected and cultured in DMEM/F12 (1:1) medium containing 10% fetal bovine serum (FBS) at 37°C in a 5% CO_2_ atmosphere. Primary adipocytes were divided into two groups. In the control group, the cells were cultured with medium only. In the NW group, the cells were treated with NW (100 μM) for 36 h.

The HepG2 cell line was purchased from the National Infrastructure of Cell Line Resource (China) and cultured in Dulbecco’s modified Eagle’s medium (DMEM), containing 10% FBS and 100 U/mL penicillin–streptomycin at 37°C in a 5% CO_2_ atmosphere. To assess NW’s effect on CSE expression, HepG2 cells were treated with different doses of NW (0, 10, 20, 40, 75, and 150 μM) for 24 h.

### Kidney I/R Model Establishment and Experimental Design

For establishing the kidney I/R model, rats were anesthetized with pentobarbital sodium (40 mg/kg, i.p.), which was followed by making abdominal incision. Bilateral renal arteries were separated, and blood flow was occluded with a vascular clip. The color of the kidney turned pale during ischemic induction. 1h after ischemia, the vascular clips were removed and the abdominal area was sutured. After reperfusion for 24 h, 2 weeks, and 4 weeks, respectively, rats were anesthetized and sacrificed, and the kidney tissue and serum were collected for further analysis.

Based on the established model, the following experimental groups were formed to analyze the effect of NW on kidney I/R:

Group I (sham): rats that only underwent separation of vascular without the obstruction of renal arteries.

Group II (I/R): rats subjected to kidney ischemia and reperfusion for indicated time (24 h, 2 weeks, or 4 weeks).

Group III (I/R + NW): to determine the role of NW in acute kidney I/R injury, SD rats were treated with NW (42 mg/kg, i.g.) at 2 h prior to the onset of kidney ischemia and three times in 24 h (8 h interval) after reperfusion. 30 min after the final treatment, the rats were sacrificed. For detecting the role of NW in long-term kidney I/R injury, SD rats were orally administered NW (42 mg/kg) at 2 h prior to the onset of kidney ischemia and one time per day for 2 or 4 weeks after reperfusion.

### Kidney Tubules’ Injury Score

Tubular injury was evaluated based on a semiquantitative scale as it was previously described. Briefly, each cortical tubule showing epithelial cell necrosis and brush border loss was assigned a score of 0 for normal, 1 for loss of brush border or cell necrosis in < 25% of tubular cells, 2 for cell necrosis in 25–50% of tubular cells, 3 for cell necrosis in 50–75% of tubular cells, and 4 for cell necrosis in > 75% of tubular cells. Two fields of magnification of ×200 per animal were examined.

### Biotin Switch Assay

The assay was performed as described with modification (Du et al., 2019). Briefly, kidney tissues were homogenized in RIPA lysis buffer (Sigma-Aldrich, United States). The homogenates were centrifuged at 14,000 rpm (4°C) for 15 min. The supernatant was collected, and protein was quantitated by the BCA assay. The primary anti-*β*-actin antibody (2 μg) was added into protein lysis (1 mg/ml) containing Protein A beads (Sigma-Aldrich) and then incubated overnight at 4°C. Beads were washed with PBS three times and then blocked with HEN buffer containing 2.5% SDS and 20 mM methyl methanethiosulfonate (MMTS) at 50°C for 20 min. MMTS was removed by precipitating proteins with acetone at −20°C for 20 min. After acetone removal, protein was resuspended in HENS buffer (containing 1% SDS). A small part of the resuspending protein was separated as input, and 4 mM biotin-HPDP was added into the remaining resuspending protein for incubation for 4 h at room temperature. Biotinylated protein was pulled down by streptavidin magnet beads, eluted by SDS-PAGE loading buffer, and subjected to western blot analysis.

### Blood Urea Nitrogen and Serum Creatinine Detection

Blood samples were centrifuged for 10 min at 5,000 rpm, and serum samples were stored at −20°C until measurement. The contents of blood urea nitrogen (BUN) and serum creatinine (Cr) were determined with an automatic clinical analyzer in the Department of Laboratory Medicine of Peking University Third Hospital.

### Blood Pressure Detection

The daily blood pressure was measured by the tail-cuff method (BP-98A, Softron, Japan), which was non-invasive and did not require surgery. All the rats were acclimatized to the restrainer and underwent tail-cuff inflation for a week before recording blood pressure.

For telemetry measurements, rats were anesthetized with pentobarbital sodium (40 mg/kg, i.p.), and the telemetric BP radio-transmitter (DSI, Minnesota, United States) was implanted by common carotid artery intubation. Blood pressure was measured from the common carotid artery by using a transducer and a computer (DSI, Minnesota, United States).

### H&E Staining

The kidney and femoral artery were fixed in 4% paraformaldehyde, embedded in paraffin, and sliced into 6 μm thick cross-sections. Sections were preheated to 65°C in a vacuum oven for 30 min and then deparaffinized immediately. Paraffin sections were stained with hematoxylin and eosin (H&E) and examined using light microscopy.

### Immunohistochemical Staining

Paraffin sections were incubated with 3% hydrogen peroxide to block endogenous peroxidase activity. To augment the expression of antigen in tissues, tissue sections underwent antigen retrieval. Antigen retrieval was performed for 30 min at 97°C in citrate buffer (pH 6.0, Abcam, ab93678), and sections were cooled to room temperature (RT). Thereafter, tissue sections were blocked with 10% BSA for 1 h and incubated with primary antibodies CSE (ab136604, Abcam, 1:200 dilution) or cleaved caspase 3 (ab214430, Abcam, 1:200 dilution) at 4°C overnight. After washing in phosphate-buffered saline (PBS) three times each for 5 min, tissues were incubated with horseradish peroxidase–conjugated anti-rabbit or anti-mouse IgG polymer as a secondary antibody for 1 h at room temperature according to the manufacturer’s instructions. Antibody labeling using IHC was performed with the 3,3′-diaminobenzidine (DAB) kit. Reacted for 5 min and the samples were rinsed with the buffer solution, counter-stained with hematoxylin, and sealed with resin mount.

### Dihydroethidium Staining

Freshly prepared frozen aortic sections were incubated with 5 μmol/L fluorescent dye dihydroethidium (DHE) at 37°C for 30 min in a humidified chamber and protected from light. Fluorescence images were captured under a TCS SP5 confocal microscope (Leica, Germany).

### Western Blot

The total protein of cells or tissues was quantified by the BCA assay. Protein samples were separated by SDS-PAGE and transferred to polyvinylidene fluoride membranes. Then, the PVDF membranes were blocked by 5% defatted milk for 1 h and incubated with the primary antibody at 4°C overnight. After washing and incubating with the horseradish peroxidase–conjugated secondary antibody for 1 h at room temperature, the results were detected using the chemiluminescence kit.

### RNA Extraction and qRT-PCR

Total RNA from tissues was extracted using TRIZOL reagent (Invitrogen) according to the manufacturer’s instructions. RNA was reverse transcribed into cDNA by using the cDNA synthesis kit (Thermo Scientific, K1622). The real-time PCR was performed in a final volume of 20 μL, which contained 10 μL of 2 × SYBR mixture, 1 μL of forward and reverse primers, respectively, 4 μL of template cDNA, and 5 μL of RNase-free H_2_O. A sample without cDNA was subjected to an identical protocol as a negative control. The PCR amplification was accomplished with initial denaturation at 95°C for 10 min, followed by 40 cycles at 95°C for 15 s and 1 min of primer annealing and extension at 60°C. The relative expression of target genes was normalized to that of 18S rRNA and analyzed by the 2^−ΔΔCT^ method. The primer sequences used for qRT-PCR are provided in Supplementary Table S1.

### Statistical Analysis

All observations were confirmed by at least three independent experiments. The data are presented as mean ± SD. Statistical significance of differences between groups was analyzed by *t*-test or by one-way analysis of variance (ANOVA) when more than two groups were compared. *p* values <0.05 were considered statistically significant.

## Results

### NW Directly Interacts With CSE

**FIGURE 1:**
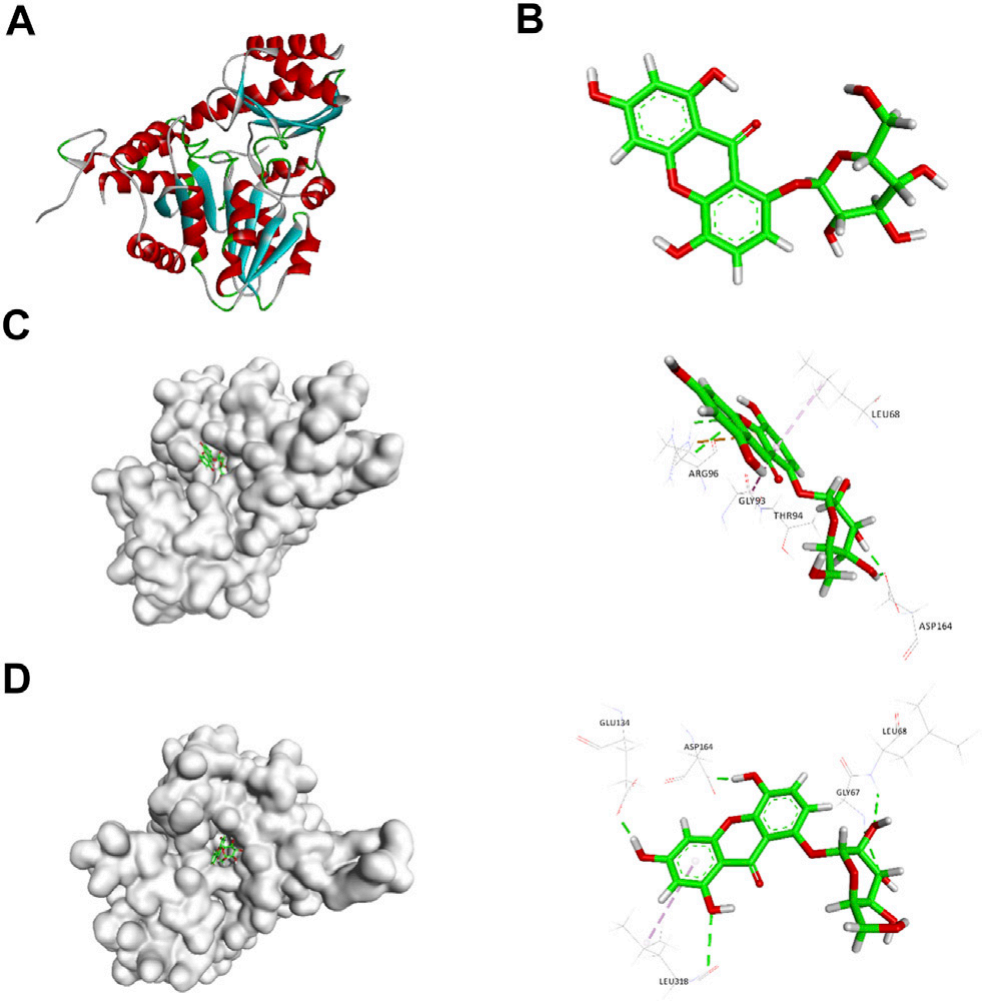
Schematic diagram of CSE **(A)** and NW **(B)** structures. Schematic diagram of CSE and NW interactions **(C, D)** by computer simulation and dynamics testing.

Based on the 3D structure of human CSE (Figure 1A) from the Protein Data Bank, we screened a natural small molecule norswertianolin (NW) (Figure 1B) from the Chinese Natural Products Database (CNPD), which had high affinity binding to CSE using computer molecular docking technology. Meanwhile, analysis showed two possible models of NW interaction with CSE (Figures 1C,D). The first model has six binding sites: Leu68, Arg96, Gly93, Thr94, Asp164, and Ser186 (Figure 1C), and the second model has five binding sites: Gly67, Leu68, Glu134, Asp164, and Leu318 (Figure 1D). Thus, Leu68 and Asp164 of CSE may be the essential binding sites for its interaction.

**FIGURE 2:**
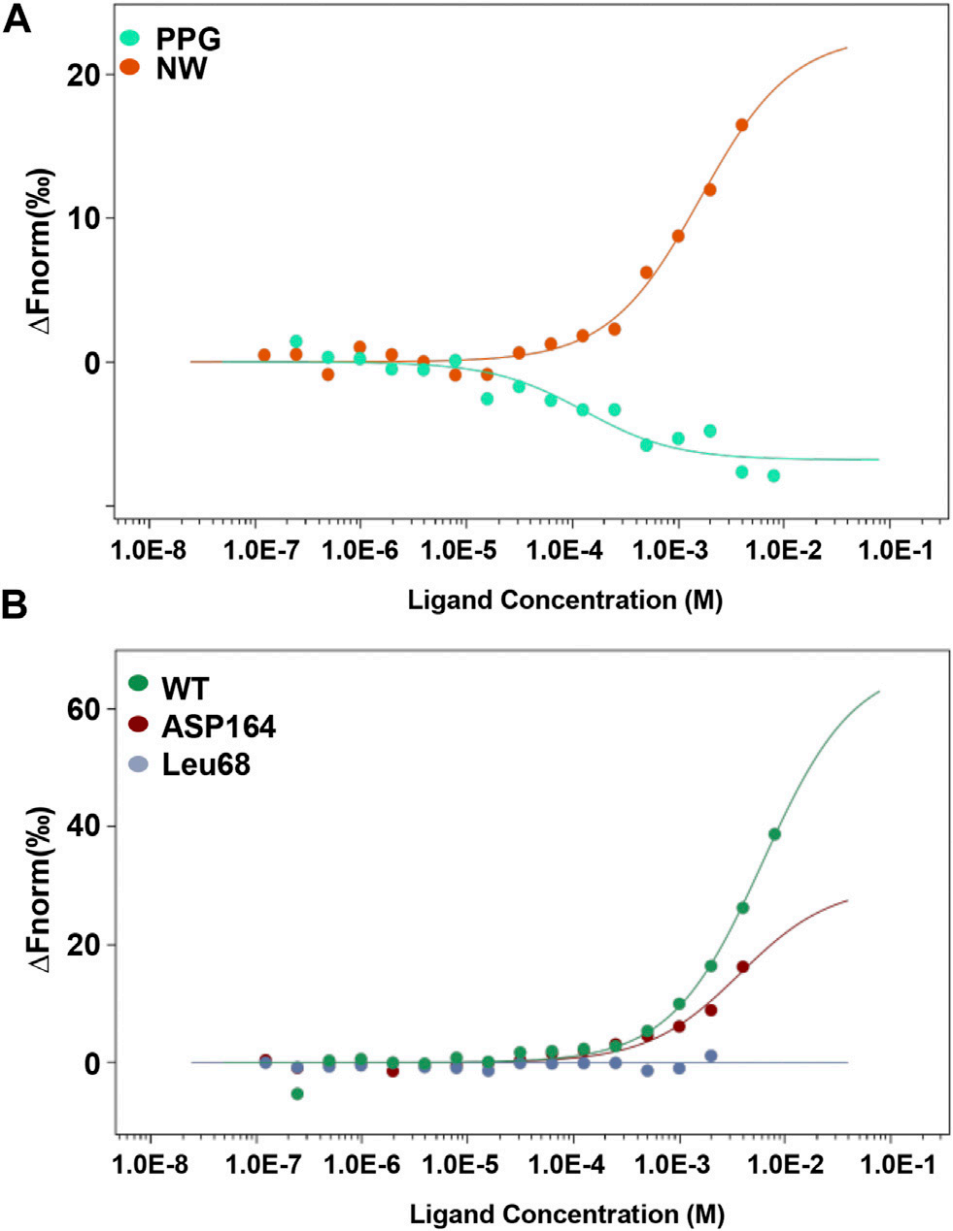
NW binding to CSE. Kinetics of NW binding to CSE assayed by microscale thermophoresis (MST) **(A)**. CSE inhibitor PPG, as a positive control. Binding kinetics of NW with CSE mutation (Leu68 site and Asp164 site) **(B)**.

To confirm the interaction between CSE and NW, we analyzed the kinetic curve of NW binding to CSE using microscale thermophoresis (MST) technology, with CSE-green fluorescent protein (GFP) fusion protein as the receptor. We first confirmed NW veritable binding to CSE with kd = 1.6 ± 0.33 μM, with CSE inhibitor–PPG binding (kd = 0.12 ± 0.05 μM) as positive control (Figure 2A). The kinetics parameters suggested that NW also had similar affinity with CSE. To identify the hydrogen bond site, we repeated the binding assay using mutant CSE-GFP protein and demonstrated that Leu68 mutation but not Asp164 abolished the interaction between NW and CSE (Figure 2B), suggesting Leu68 is the key hydrogen bond binding site of NW binding to CSE.

### NW Enhances CSE Activity for H_2_S Generation

**FIGURE 3:**
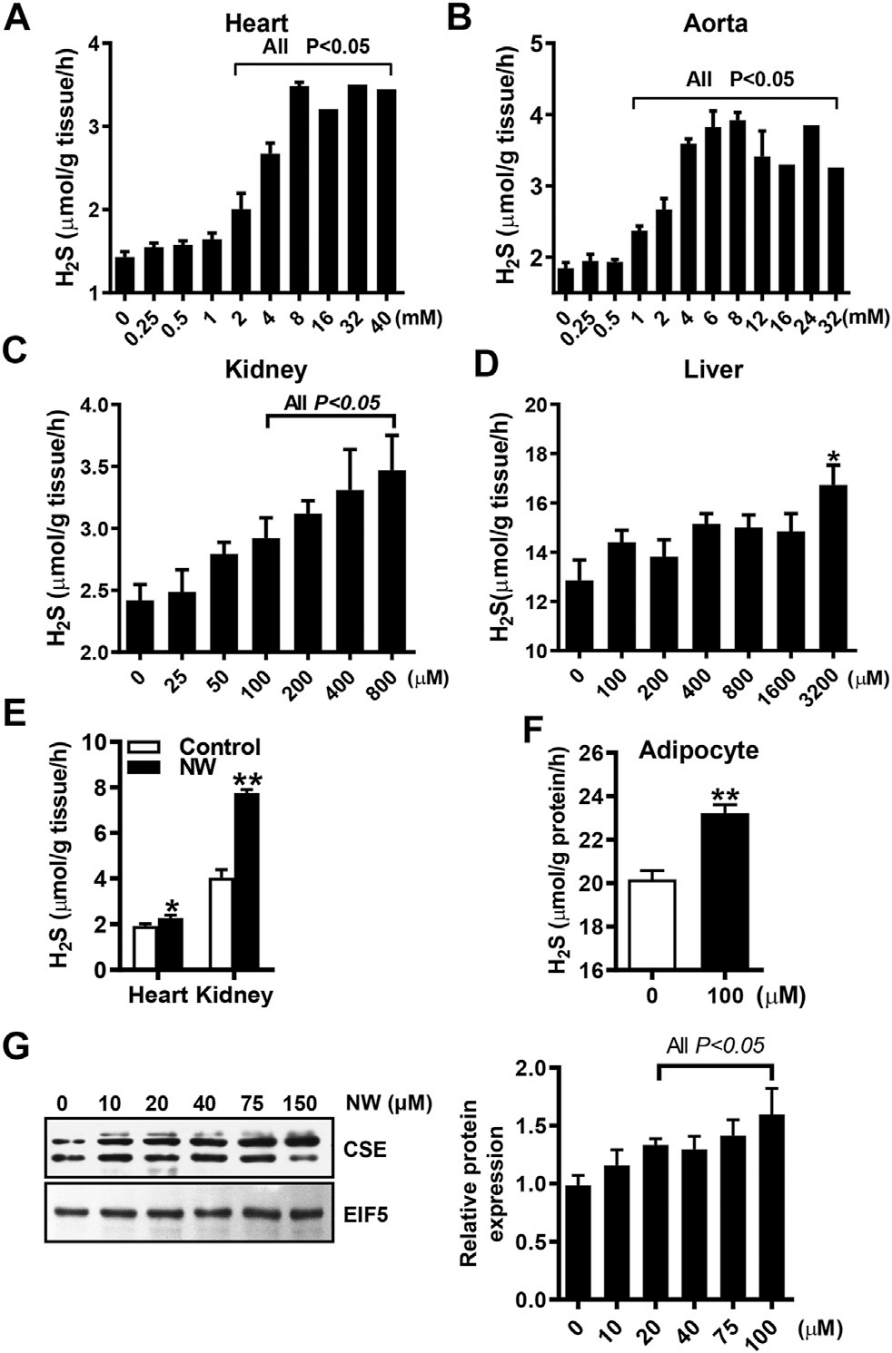
NW improved CSE/H_2_S production. L-Cysteine and pyridoxal phosphate and indicated concentration of NW are added to tissue homogenates. H_2_S production in the heart **(A)**, aorta **(B)**, kidney **(C)**, and liver **(D)** was measured, N = 4. SD rats were subcutaneously injected with NW 4.4 mg/kg/day for 1 week. H_2_S production in the heart and kidney was detected **(E)**, N = 5. In primary adipocytes, the effect of 100 μM NW treatment on H_2_S production was detected **(F)**, N = 5. In cultured HepG2 cells, the effect of different concentrations of NW on the CSE protein level was detected. The left panel is representing graph, and the right panel is statistical graph **(G)**, N = 3. **p < 0.01.

To determine whether NW is functionally relevant to CSE activity, we detected the NW effect on H_2_S production in various tissue homogenates. With L-cysteine and pyridoxal phosphate, NW increased H_2_S production in the heart, aorta, and kidney (Figures 3A–C), whereas only high concentration of NW (3,200 μM) stimulated H_2_S generation in the liver (Figure 3D). Furthermore, we detected the effect of NW on H_2_S production *in vivo*. Treating with NW for 1 week, heart and kidney tissues’ H_2_S generation was significantly upregulated in comparison with that in the DMSO group (Figure 3E). In isolated primary adipocytes (only expressing CSE), 100 μM NW significantly increased H_2_S generation (Figure 3F). Consistently, in cultured HepG2 cells, high concentration of NW increased the CSE protein level (Figure 3G). These results highlight that NW indeed heightened CSE activity for H_2_S generation in isolated cells, *ex vivo* and *in vivo*.

### NW Ameliorates Kidney I/R Injury

**FIGURE 4:**
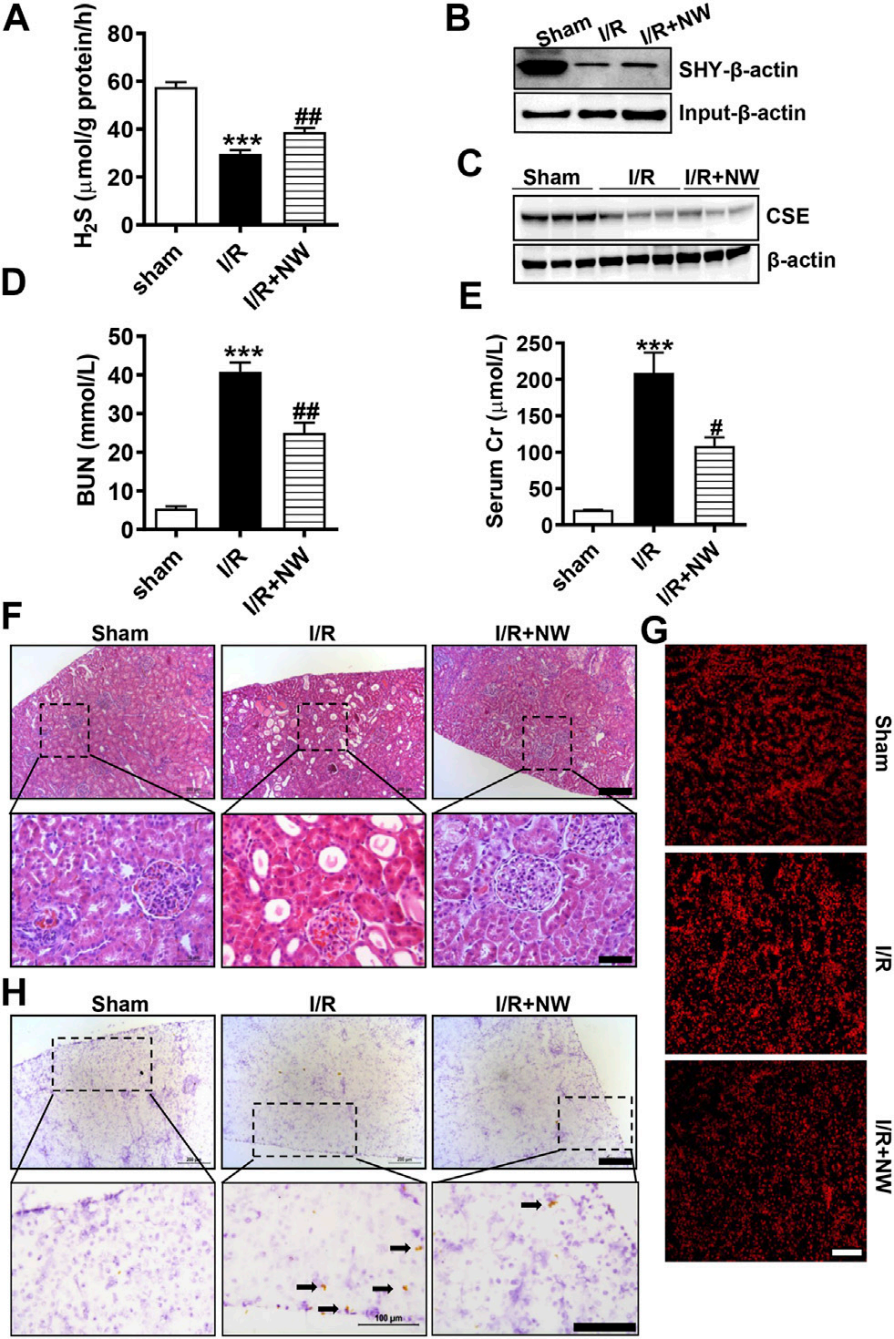
NW is protective against acute kidney ischemia–reperfusion injury. 1 h after kidney ischemia and 24 h after reperfusion, H_2_S production in the kidney was detected **(A)**. The modified biotin switch assay determined sulfhydrated *β*-actin (SHY-*β*-actin) expression **(B)**. Western blot analysis of CSE protein levels **(C)**. Blood urea nitrogen **(D)** and serum Cr (creatinine) **(E)** contents in sham, I/R, and I/R + NW groups. H&E staining showed changes of morphology in the kidney **(F)**. Upper panel: scale bar = 200 μm; lower panel: scale bar = 50 μm. DHE staining indicated the oxidative stress level **(G)**, scale bar = 200 μm. Immunohistochemical staining of cleaved caspase 3 in the kidney **(H)**. Upper panel: scale bar = 200 μm; lower panel: scale bar = 100 μm. Black arrows point out the positive cells. N = 6/group. *Versus sham, ^#^ *versus* I/R.

H_2_S plays a protective role in kidney ischemia/reperfusion (I/R) injury (Wu et al., 2015). NW also dramatically increased kidney H_2_S production (Figure 3E). Therefore, we investigated the therapeutic effects of NW on kidney I/R injury. In the acute kidney I/R injury model, H_2_S production was dramatically decreased in kidney I/R and partly recovered by NW treatment (Figure 4A). Consistently, the sulfhydrated *β-*actin (SHY-*β*-actin, a specific chemical modification by H_2_S at cysteine residue) level in the I/R kidney was lower than that in sham, which was partly rescued by NW (Figure 4B and Supplementary Figure S1A). CSE protein expression also decreased in the I/R kidney, but NW treatment did not recover it (Figure 4C and Supplementary Figure S1B). These results indicated that NW treatment increased CSE activity but not its expression in acute kidney I/R.

To investigate the effect of NW on kidney I/R injury, blood urea nitrogen (BUN) and serum creatinine (Cr)—the sensitive indicators of renal function—were measured. Compared with those in the sham group, BUN (Figure 4D) and serum Cr (Figure 4E) levels were significantly heightened in the I/R group, which were reversed by NW (Figures 4D,E). H&E staining showed obvious tubular injury as loss of brush border, cast formation, and extensive loss of tubular epithelial cells and tubular dilation in the I/R kidney. NW treatment attenuated the renal tubular injury in acute I/R (Figure 4F and Supplementary Figure S1C). Oxidative stress and cell apoptosis are the key factors of kidney damage after I/R. In keeping with pathological changes, NW also reduced I/R-stimulated ROS by DHE fluorescence intensity (Figure 4G and Supplementary Figure S1D) and apoptosis cells by cleaved caspase 3 immunohistochemical stain (Figure 4H and Supplementary Figure S1E). Taken together, NW exhibited protection against acute kidney I/R injury similar to the H_2_S donor.

**FIGURE 5:**
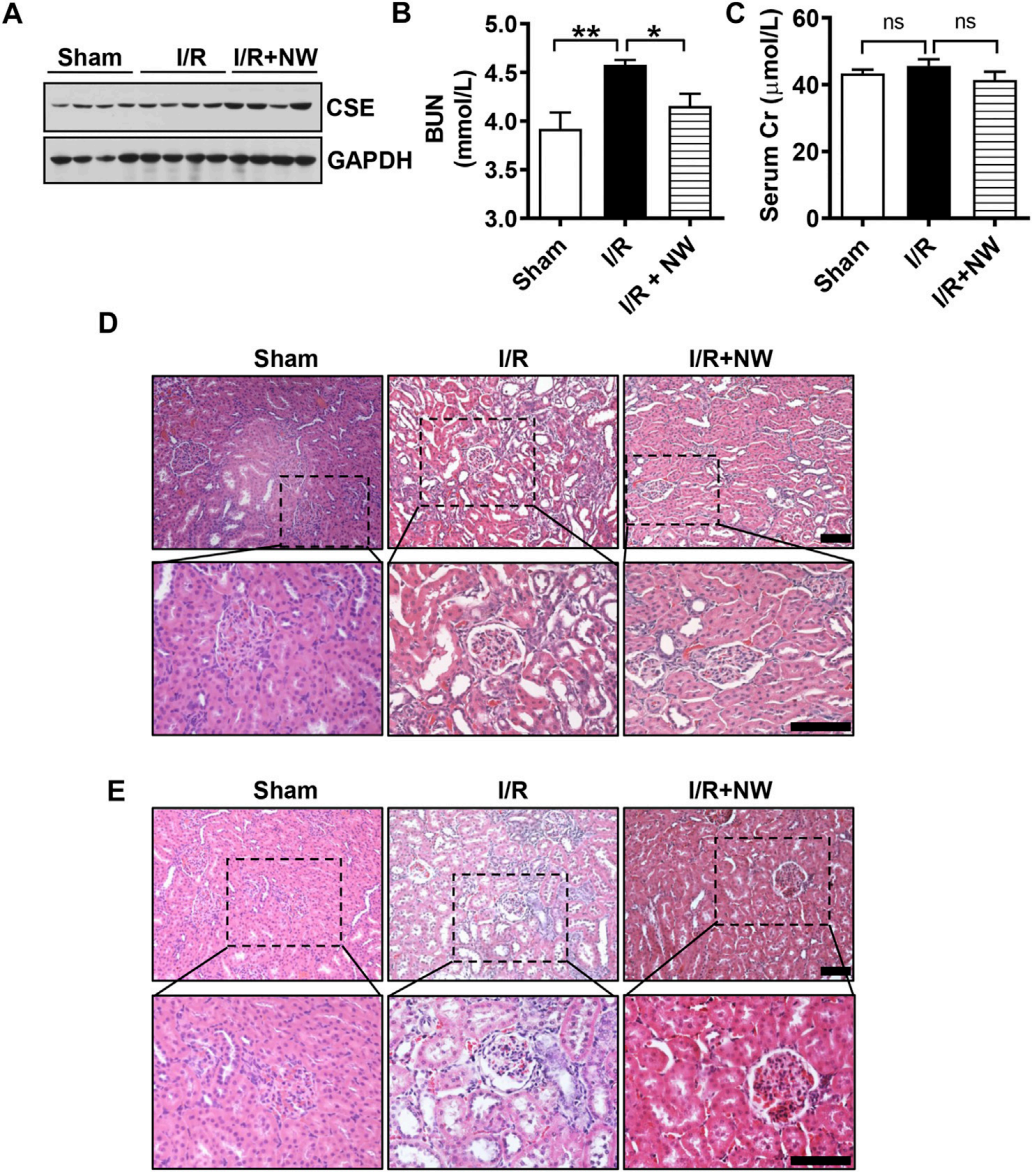
NW plays a long-term protective role in kidney ischemia–reperfusion injury. 1 h after kidney ischemia and 2 weeks after reperfusion, western blot analyzes the CSE protein level **(A)**. Blood urea nitrogen **(B)** and serum Cr (creatinine) **(C)** contents in sham, I/R, and I/R + NW groups. H&E staining of the kidney 1 h after kidney ischemia and 2 weeks after reperfusion **(D)**, scale bar = 100 μm. H&E staining of the kidney 1 h after kidney ischemia and 4 weeks after reperfusion **(E)**, scale bar = 100 μm. N = 6/group. *p < 0.05, **p < 0.01. ns: no significant difference.

To further confirm the long-term effects of NW on kidney I/R injury, we used the model of kidney ischemia (1 h)/reperfusion (2 w/4 w) injury in rats. Interestingly, NW also increased CSE protein expression after 4 weeks of kidney I/R injury (Figure 5A and Supplementary Figure S2A). NW treatment for 4 weeks also reduced BUN (Figure 5B). However, there is no statistical difference in serum Cr among all groups (Figure 5C). Histopathologic results also showed NW attenuated renal tubular damage and inflammatory cell infiltration 2 weeks (Figure 5D and Supplementary Figure S2B) and 4 weeks (Figure 5E and Supplementary Figure S2C) after reperfusion. All these results indicated that NW activated CSE and then elevated H_2_S generation to attenuate kidney I/R injury.

### NW Exhibits Anti-Hypertension, Alleviating Vascular Remodeling and Repressing Inflammation

**FIGURE 6:**
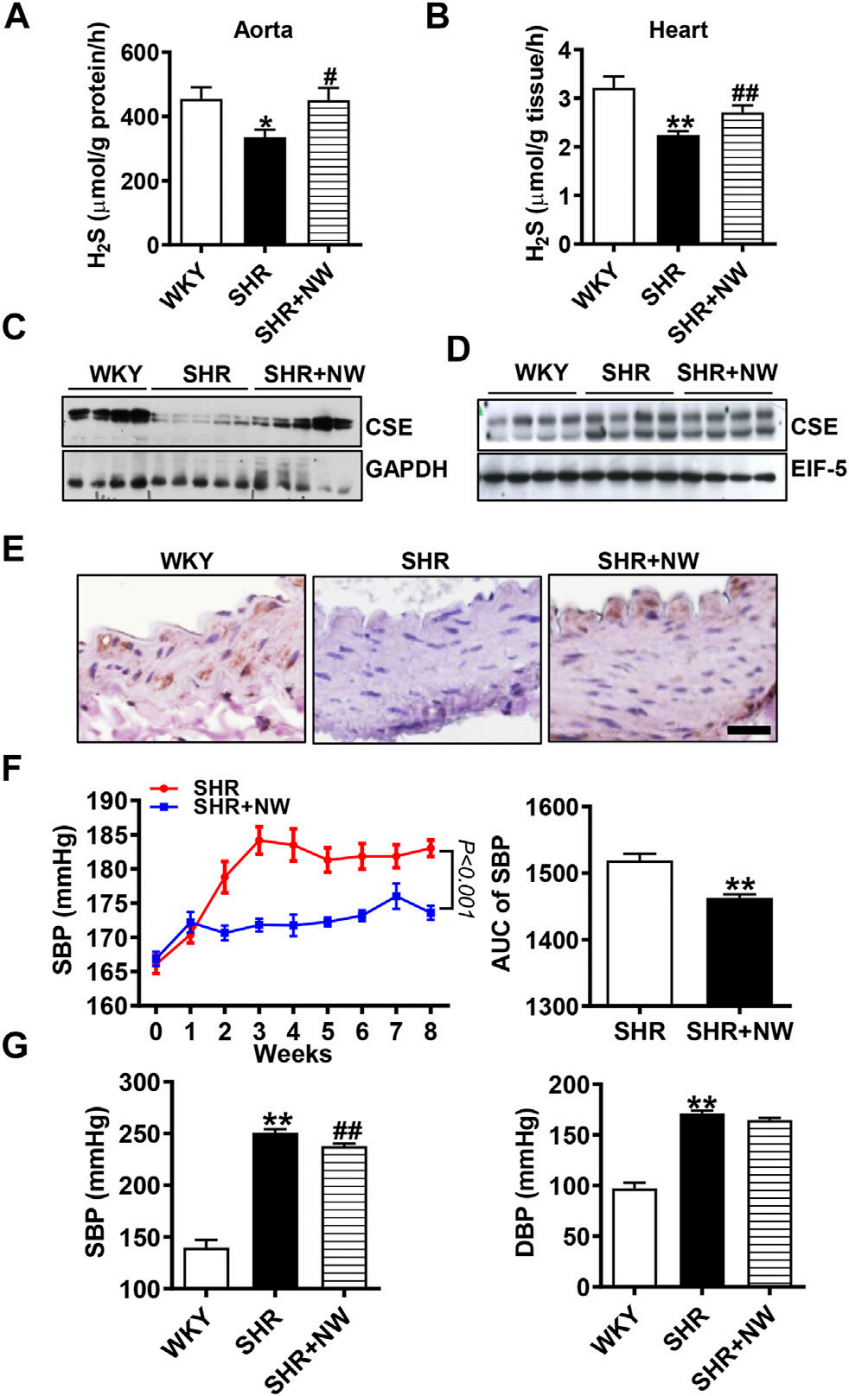
NW treatment reduces blood pressure of SHRs. H_2_S production measurement by the methylene blue assay in the aorta **(A)** and heart **(B)**. The western blot assay analyzes CSE expression in the aorta **(C)** and heart **(D)**. Immunohistochemical staining of CSE in the aorta **(E)**, scale bar = 25 μm. Blood pressure of SHRs and SHRs + NW monitored by tail arteries; the right panel is area under the curve of the left panel **(F)**. 8 weeks after NW treatment, blood pressure was monitored by telemetry measurements **(G)**. N = 10/group. *Versus WKY rats, ^#^ *versus* SHRs. *p < 0.05, **p < 0.01.

**FIGURE 7:**
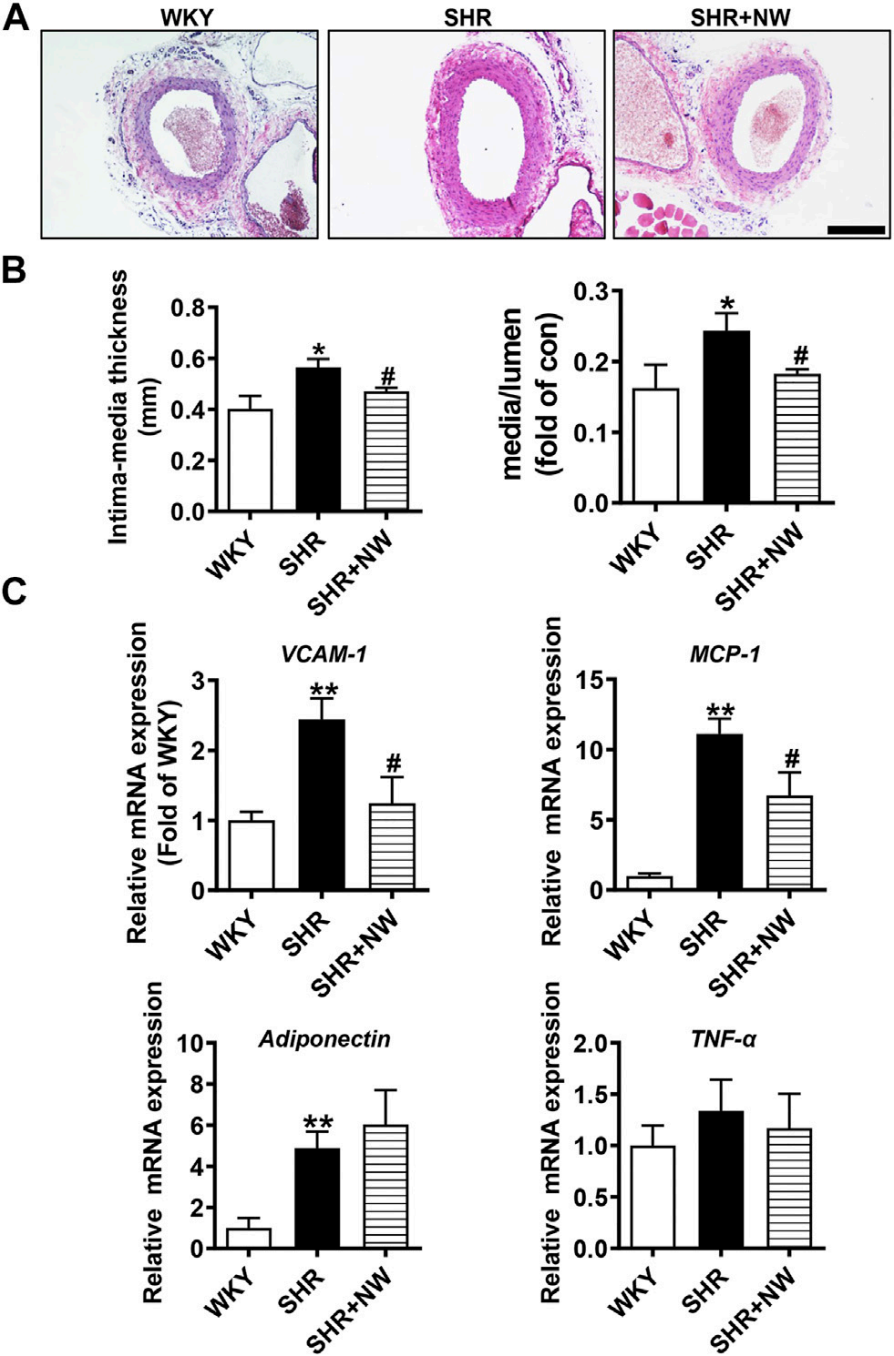
NW treatment restrains vascular remodeling and vascular inflammation in SHRs. H&E staining of the femoral artery **(A)**, scale bar = 100 μm. Femoral artery intima media thickness and ratio of media to lumen **(B)**. Real-time PCR analysis of VCAM-1, MCP-1, adiponectin, and TNF-α mRNA levels **(C)**. N = 10/group. *Versus WKY rats, ^#^ *versus* SHRs. *p < 0.05, **p < 0.01.

CSE/H_2_S plays a critical role in blood pressure regulation and vascular remodeling. Here, we also treated SHRs with NW (4.42 mg/kg/day) for 8 weeks. As H_2_S effects (Yan et al., 2004), NW treatment heightened the H_2_S production in the aorta (Figure 6A) and heart (Figure 6B) of SHRs and upregulated CSE expression in the aorta (Figure 6C and Supplementary Figure S3A). However, NW treatment did not enhance CSE expression in the heart (Figure 6D and Supplementary Figure S3B). Immunohistochemical stain also confirmed CSE expression upregulation in the aorta (Figure 6E). Systolic blood pressure (sBP) lowered after treating with NW for 2 weeks, and continuous to 8 weeks by tail artery BP measurement (Figure 6F). The lowered sBP was also confirmed by telemetry at the end of NW treatment for 8 weeks (Figure 6G). Although diastolic blood pressure also slightly reduced by NW, there is no statistical difference (Figure 6G). In line with changes of sBP, NW administration enlarged lumen diameter and reduced media thickness and media/lumen ratio in the femoral artery by H&E staining (Figures 7A,B). Mechanically, NW inhibited VCAM-1 and MCP-1 mRNA expressions but had no effect on TNF-α and adiponectin (Figure 7C). Overall, these findings revealed that NW has anti-hypertension effects, inhibiting vascular remodeling and reducing inflammation effects.

## Discussion

CSE/H_2_S plays an essential protective role in cardiovascular diseases. More and more studies focus on H_2_S donor development, design, and synthesis, such as GYY4137, SG1002, and ATB-346. Some of them had progress in phase I or II clinical trials. In most studies, CSE/H_2_S downregulated in cardiovascular diseases, and the key issue is the CSE enzyme expression and activity reduction. In the present study, we target the key enzyme of H_2_S generation—CSE in the cardiovascular system, design, and identify a natural small molecule NW which specifically binds to CSE, and the major hydrogen bond site is Leu68. Functionally, NW significantly increased CSE activity including CSE protein expression and H_2_S generation *in vitro* and *in vivo.* For evaluating the NW therapeutic possibility, NW treatment exhibited acute or long-term protection against kidney I/R by reducing the ROS level and cell apoptosis. Furthermore, NW administration attenuated blood pressure and vascular remodeling by inhibiting inflammation in the SHR model. These data highlight that NW is a novel natural chemical CSE agonist and has potential therapeutic merits for cardiovascular diseases.

The protective effect of H_2_S has been verified in various cardiovascular diseases such as hypertension, atherosclerosis, ischemic injury, and shock. NaHS is the widely used early H_2_S donor in experimental studies such as anti-hypertension (Yan et al., 2004), lowering atherosclerotic plaque (Liu et al., 2013), and alleviating I/R injury with anti-inflammation and anti-apoptosis (Wu et al., 2015). However, this kind of donor releases H_2_S too fast to maintain H_2_S concentration. Other H_2_S donors, which release H_2_S gently, tend to produce secondary products or need to be in high dose, leading to liver and kidney toxicity (Wu et al., 2016; Powell et al., 2018). Although SG1002 and ATB-346 are safe, the efficiency for cardiovascular diseases by long-time observation still needs to be confirmed. In comparison with the synthetic chemical molecular compounds, natural products are safer and druggable. Here, we designed and confirmed a natural chemical product norswertianolin, bound it to CSE, enhanced its protein expression and activity for H_2_S, and exhibited protection against I/R injury and hypertension. More interestingly, NW in high dose (up to 400 mg/kg/day) has still low toxicity (did not elevate serum GST, GLT, BUN, and Cr; data not shown). Thus, NW has the potential translational merit and druggability.

CSE is the major enzyme of H_2_S generation in the cardiovascular system. So, H_2_S generation is dependent on CSE activity. Here, we demonstrated that, in heart, aorta, and kidney homogenates, high-dose NW increased H_2_S generation, while H_2_S generation is not significantly upregulated in the liver homogenate after NW administration. We speculate the reason is that both CSE and CBS were expressed enriched in the liver led to the high production of H_2_S in the basal condition without NW treatment. CSE deficiency of vascular smooth muscle cells and CD4^+^ T cells lost beyond 80% H_2_S generation (Yang et al., 2010; Cui et al., 2020). Supplementation of H_2_S donors also increased the CSE protein level in a dose-dependent manner (Li et al., 2016). Consistently, our results showed heart and kidney H_2_S generation also increased when rats were gavaged with NW. *In vitro*, NW pretreatment not only enhanced H_2_S production in isolated adipocytes but also upregulated the CSE protein level in HepG2 cells. We speculate that NW treatment increases CSE activity and promotes H_2_S production, which in turn enhances CSE expression. Additionally, the spatial conformation of CSE protein changed after interacting with NW, which may make CSE protein difficult to degrade. Overall, our results demonstrated that NW supplementation increases CSE activity and promotes H_2_S generation.

Kidney ischemia/reperfusion injury is a major complication of kidney tumor resection, transplantation, and hypovolemic shock. In kidney I/R models, both CSE and CBS expressions were downregulated in the kidney (Han et al., 2015). Meanwhile, serum and kidney H_2_S levels markedly reduced (Xu et al., 2009). CSE deletion aggravated kidney damage and mortality when subjected to kidney I/R (Bos et al., 2013). CSE or CBS inhibitor supplementation also deteriorated kidney I/R injury (Tripatara et al., 2008; Han et al., 2015). These results indicated impairment of endogenous H_2_S generation contributed to kidney I/R injury. Our results showed NW increased kidney CSE expression and H_2_S production in acute kidney I/R models. Exogenous H_2_S supplementation or H_2_S donor’s administration plays protective effect on kidney ischemia/reperfusion injury. Mechanistically, H_2_S lowers the metabolic rate, decreases oxidative stress, and reduces cell apoptosis to attenuate kidney I/R injury (Bos et al., 2009; Tripatara et al., 2009; Hunter et al., 2012; Azizi et al., 2016). In agreement with previous studies, NW pretreatment reduced ROS production and cleaved caspase 3 expression to improve kidney function in acute kidney I/R models. Due to enhanced CSE activity and endogenous H_2_S production, NW has endurable protection in kidney I/R models.

The H_2_S level is closely related to hypertension. In humans, plasma H_2_S concentration is lower in hypertension patients than in normotension people (Sun et al., 2007). CSE/H_2_S reduction caused maternal hypertension and placental abnormalities in preeclampsia (Wang et al., 2013). In hypertension animal models, endogenous H_2_S generation was also reduced (Zhong et al., 2003; Ahmad et al., 2012; Al-Magableh et al., 2015). CSE-deficient mice exhibited severe endothelial dysfunction and hypertension; H_2_S donor supplementation lowered blood pressure of CSE knockout mice. Therefore, the H_2_S donor improved CSE/H_2_S expression in hypertensive animals (Zhong et al., 2003; Yan et al., 2004; Al-Magableh et al., 2015). Here, our results confirmed CSE/H_2_S reduction in the aorta and heart of SHRs compared with WKY rats. NW upregulated CSE protein and H_2_S generation in the aorta and heart and lowered blood pressure of SHRs. Exposure to hypertension and its risk factors, VSMCs switched systolic phenotype into synthetic phenotype, which induced vascular wall thickening, lumen stenosis, and vascular remodeling (Brown et al., 2018). H_2_S inhibits VSMC proliferation and promotes VSMC apoptosis to reverse vascular remodeling (Du et al., 2004; Tian et al., 2021). Similarly, NW reduced the ratio of media/lumen and vascular remodeling, all of which contributed to the pathogenesis of hypertension. Vascular chronic inflammation plays a crucial role in the pathophysiology of hypertension (Rodriguez-Iturbe et al., 2017). Monocyte recruitment is the key factor in the development of vascular inflammation, followed by adhesion molecules (VCAM-1 and ICAM-1), inflammatory factors (TNF-α and IL-6), and chemokines (CCL2, CXCL5, CXCL8, and CXCL10) released during hypertension (Martynowicz et al., 2014; Mehaffey and Majid, 2017). H_2_S administration decreased ICAM-1 expression and inhibited release of inflammatory factors (IL-1β, TNF-α, and IL-6) (Pan et al., 2011; Mani et al., 2014). Our study confirmed that NW like H_2_S also reduced inflammatory factor expression in the aorta of SHRs.

In conclusion, we firstly screen a new natural small molecule compound NW which binds to CSE. Functionally, NW acts as a CSE activator, improving H_2_S generation both *in vivo* and *in vitro* to alleviate kidney I/R injury and hypertension of SHRs. NW might act as a novel therapeutic selection for I/R diseases and hypertension.

## Data Availability

The raw data supporting the conclusions of this article will be made available by the authors, without undue reservation.
