# Supplementary material for: Norswertianolin Promotes Cystathionine γ-Lyase Activity and Attenuates Renal Ischemia/Reperfusion Injury and Hypertension
Source: Front Pharmacol. 2021 Jul 14;12:677212. doi: 10.3389/fphar.2021.677212 (PMC8317460; doi:10.3389/fphar.2021.677212)
Supplement: Supplementary file 1 [file DataSheet1.docx]

Supplementary Material

**Supplementary table 1.** **List of oligonucleotide primer pairs used in qRT-PCR and analysis.**

| Target Gene | Forward primer (5'-3') | Reverse primer(5'-3') |
| --- | --- | --- |
| VCAM-1(R) | GGAAATGCCACCCTCACCTT | TCCAGGGGAGATGTCAACAGT |
| MCP-1(R) | TCTCTTCCTCCACCACTATG | ATGAGTAGCAGCAGGTGAGT |
| TNF-α(R) | CCCCTTTATCGTCTACTCCT | TGTGTTTCTGAGCATCGTAG |
| Adiponectin(R) | AGTCTGGCTCCAAGTGTATG | GCTCTGAATTAGTGGCAGTC |

R: Rat


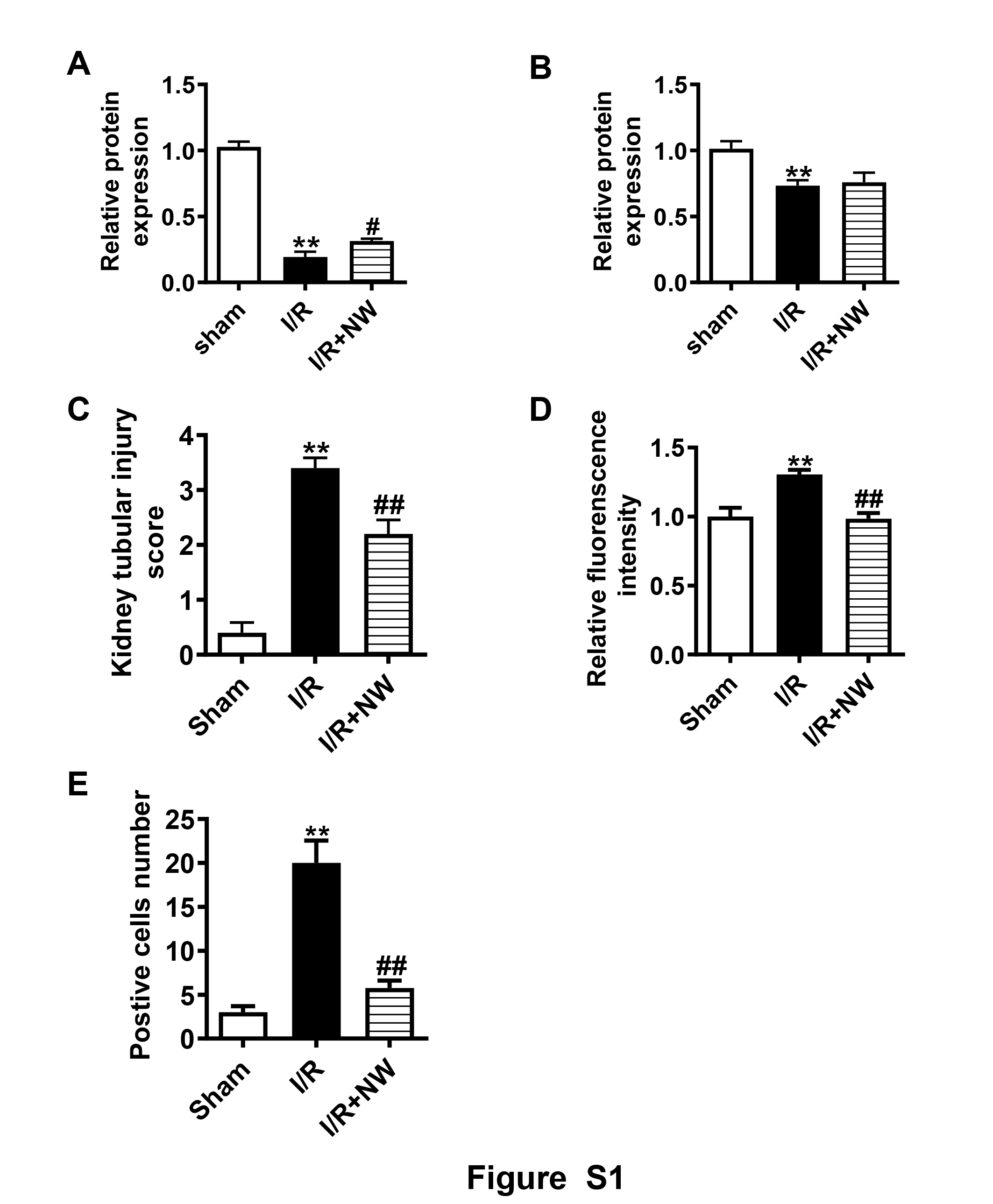


**Figure S1 NW protect against acute kidney I/R injury.** Quantitative analysis of relative protein level of SHY-β-actin (A) and CSE (B) in kidney, while subjecting to I/R and treating with NW. After NW treatment, quantitative analysis of kidney H&E staining (C), DHE staining (D) and cleaved-caspase 3 staining (E). * *P*<0.05, ** *P*<0.01. * *VS* Sham; # *VS* I/R.


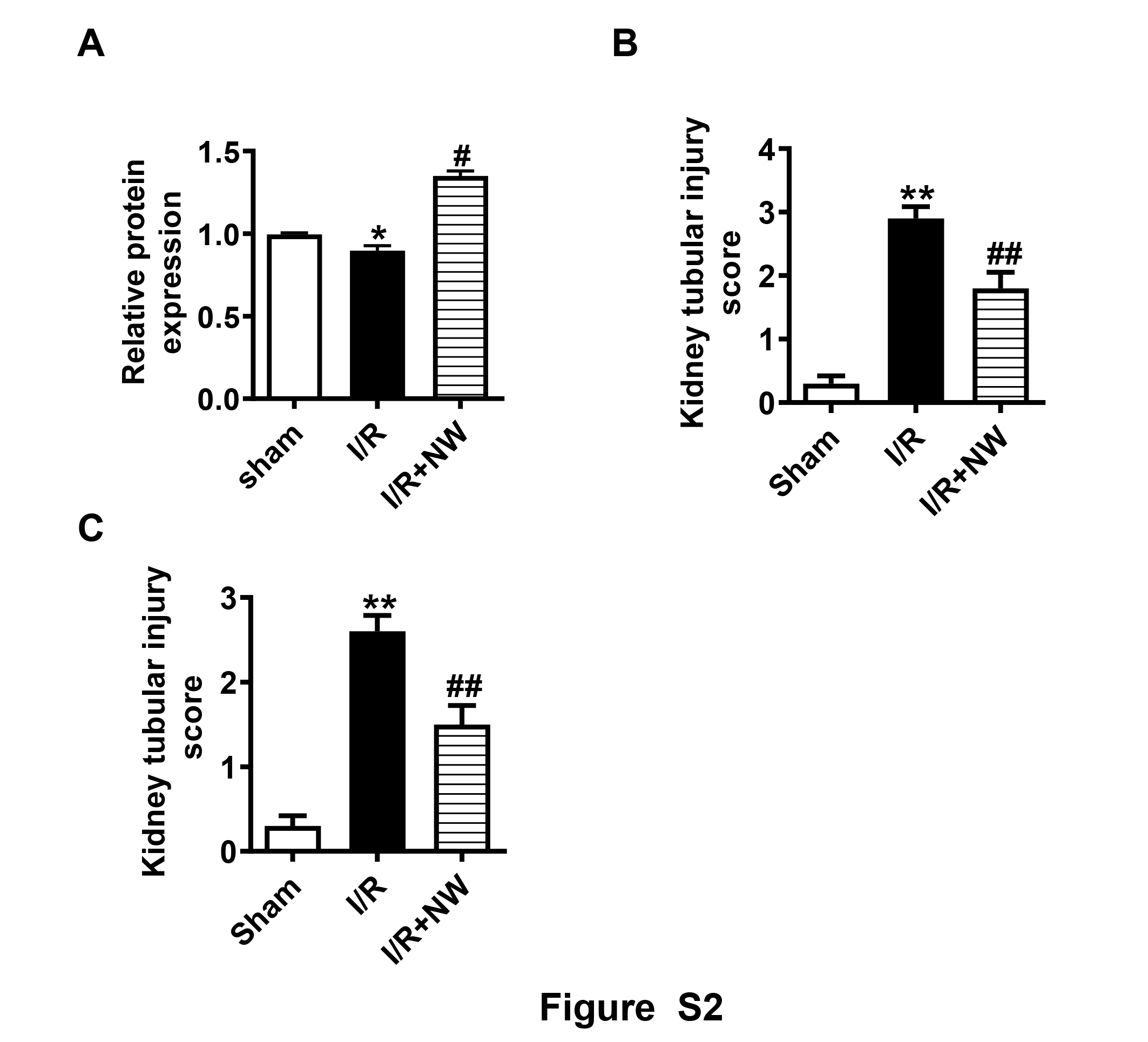


**Figure S2 NW protect against long-term kidney I/R injury.** Quantitative analysis of relative protein level of CSE (A) in kidney, while subjecting to I/R and treating with NW. After NW treatment, quantitative analysis of kidney H&E staining after ischemia 1 hour - reperfusion 2 weeks (B) and ischemia 1 hour - reperfusion 4 weeks (C). * *P*<0.05, ** *P*<0.01. * *VS* Sham; # *VS* I/R.


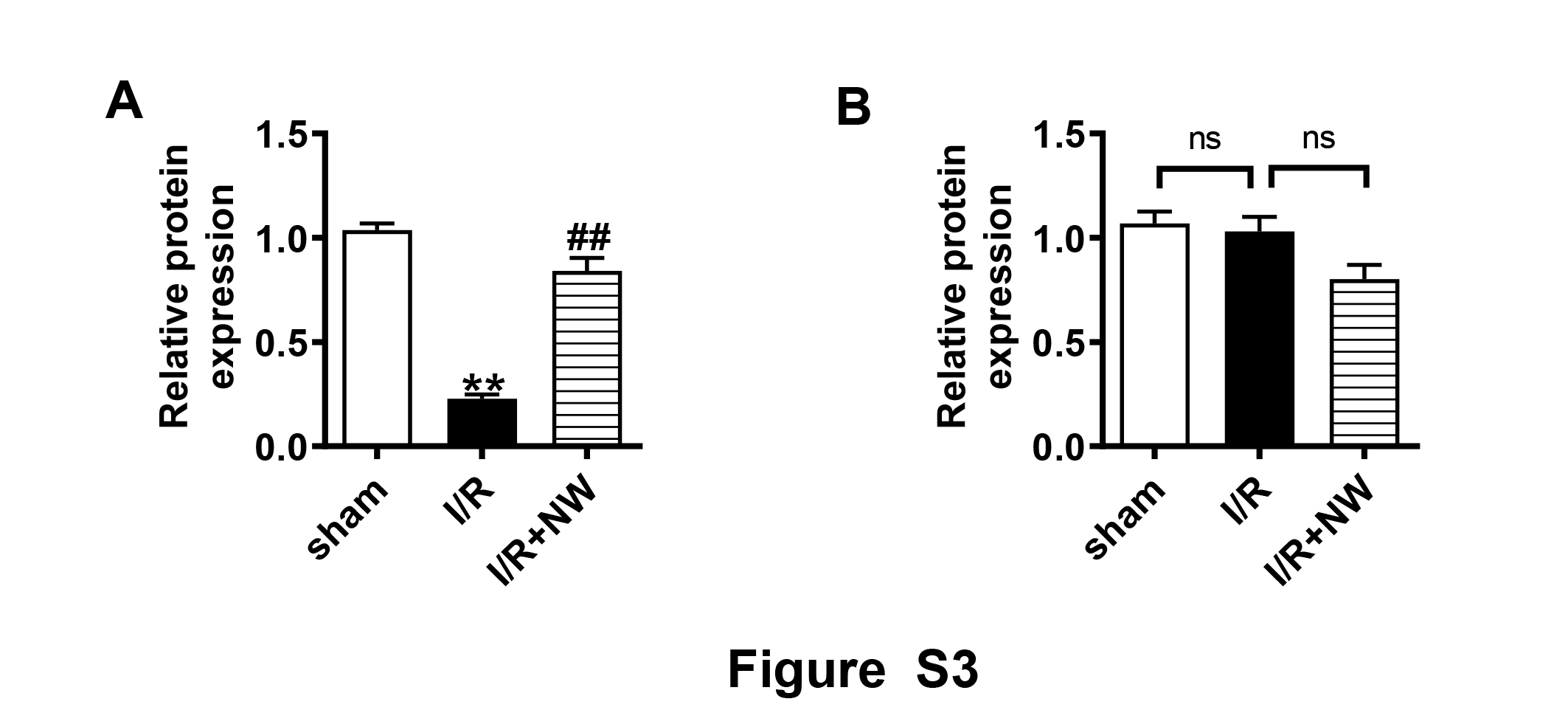


**Figure S3 CSE expression in aorta and heart after NW treatment in SHRs.** Quantitative analysis of CSE protein expression in aorta (A) and heart (B). ** *P*<0.01. * *VS* Sham; # *VS* I/R. ns: no significant difference.
